# Pathways to emotional closeness in neonatal units – a cross-national qualitative study

**DOI:** 10.1186/s12884-016-0955-3

**Published:** 2016-07-19

**Authors:** Renée Flacking, Gill Thomson, Anna Axelin

**Affiliations:** School of Education, Health and Social Studies, Dalarna University, 79188 Falun, Sweden; Maternal and Infant Nutrition and Nurture Unit (MAINN), School of Community Health and Midwifery, University of Central Lancashire, Preston, UK; Department of Nursing Science, University of Turku, Turku, Finland

**Keywords:** Closeness, Emotion, Infant, Neonatal, Parents, Thematic analysis

## Abstract

**Background:**

Research shows evidence for the importance of physical and emotional closeness for the infant, the parent and the infant-parent dyad. Less is known about how, when and why parents experience emotional closeness to their infants in a neonatal unit (NU), which was the aim of this study.

**Methods:**

A qualitative study using a salutogenic approach to focus on positive health and wellbeing was undertaken in three NUs: one in Sweden, England and Finland. An ‘emotional closeness’ form was devised, which asked parents to describe moments/situations when, how and why they had felt emotionally close to their infant. Data for 23 parents of preterm infants were analyzed using thematic networks analysis.

**Results:**

A global theme of ‘pathways for emotional closeness’ emerged from the data set. This concept related to how emotional, physical, cognitive and social influences led to feelings of emotional closeness between parents and their infants. The five underpinning organising themes relate to the: Embodied recognition through the power of physical closeness; Reassurance of, and contributing to, infant wellness; Understanding the present and the past; Feeling engaged in the day to day and Spending time and bonding as a family.

**Conclusion:**

These findings generate important insights into why, how and when parents feel emotionally close. This knowledge contributes to an increased awareness of how to support parents of premature infants to form positive and loving relationships with their infants. Health care staff should create a climate where parents’ emotions and their emotional journey are individually supported.

## Background

Physical closeness in a Neonatal Unit (NU) ranges from skin-to-skin contact between parent-infant to parents being in the unit but not in physical contact with their infant(s). Similarly, emotional closeness ranges from strong and consistent to more distant feelings of love, care, affection and/or connection between the parent and the infant. Although ‘physical closeness’ may facilitate ‘emotional closeness’ and vice versa, there may also be a disconnection between them e.g. parents can be physically close but feel emotionally detached, or even be physically remote but still feel emotionally connected [1]. Research shows evidence for the importance of physical and emotional closeness for the infant, the parent and the infant-parent dyad [2] but less is known about why, when and how parents experience emotional closeness.

When an infant is born preterm (<37 gestational weeks) or in need of neonatal care, the parents are usually separated from their infants soon or directly after birth. The enforced parent-infant separation, often attributed to the complex technological support for the infant’s viability, can lead to parents feeling: disconnected from their infant [3, 4], high levels of anxiety and stress [5] and less confident in the parental role [3, 6].

Whilst poor and restricted staff-parent interactions and communication can exacerbate parents’ sense of isolation from their preterm infants [7], it has also been suggested that parents’ negative emotions and experiences associated with prematurity or infant illness can lead them to withdraw physically and emotionally; thereby handing over the care of their infants to staff [3, 8, 9]. A recent meta-analysis [10] was undertaken to explore mother-infant interactions and relationships within preterm and full-term populations. The results revealed that during the first six months post birth, mothers of preterm infants demonstrated less positive interaction behaviors with their infants than mothers of term infants [10]. Furthermore, emerging evidence suggests that care practices that support parent-infant physical and emotional closeness, i.e. kangaroo care, decreases the prevalence of maternal depression similar to levels reported in mothers of full-term infants [11, 12]. Studies undertaken with fathers and their preterm infants have also identified that early contact can reduce stress and anxiety [13] and facilitates the attainment of the paternal role [14].

Goulet and colleagues [15] describe how physical and emotional closeness (through vocalisations, visual contact, touch and other sensori-motor interactions) are crucial to the establishment of the parent-infant relationship. Other research highlights how close physical and emotional contact facilitates the development of positive parent-infant relationships, and can enhance the parent’s confidence and capabilities in providing care for their newborn [16–18]. However, to date there has been little exploration into when, how and why parents experience emotional closeness with their infants in the neonatal environment.

The aim of this study was to explore parents’ feelings of emotional closeness when their infants are cared for in a NU. We adopted a salutogenic approach [19, 20], an orientation that focusses on how and why individuals remain physically and mentally healthy, despite enduring conditions of stress and adversity. While previous research has tended to explore the emotional disconnection between parents-infants in a NU context, we considered that a focus on positive experiences of emotional closeness would illuminate new findings. Furthermore, as the data was collected from three different countries (i.e. Sweden, England and Finland), the findings offer important insights into how, when and why parents experience emotional closeness in different contexts.

## Methods

### Design and setting

This study was conducted with a qualitative design undertaken in three NUs in Sweden, England and Finland. The Swedish NU was a level 2 unit with 14 cots. Parents stayed in their own room next to the room of the infant during the intensive care phase. When the infant no longer required ventilation, the infant was transferred with the parent to a single room, where the parents could stay with their infant for the rest of the hospital stay. In England the study was conducted in a level 3 unit with 27 cots. The unit had 8 beds on the antenatal ward which were used for mothers whose infant was admitted to the NU. The NU also had 2 overnight rooms which were used if an infant was very sick or for parents to stay with their infant prior to their discharge. In Finland, the NU was a level 3 unit with 18 cots in seven rooms accommodating two to four infants per room. There were no limitations for parental presence in the NU but only one room was available for pre-discharge overnight stays for families.

### Data collection

An emotional closeness form was devised which asked parents to describe their experiences of emotional closeness to their infant. At the top of the form there were brief instructions as to what information was required: *‘Please can you describe or record any times in your own words when you have felt emotionally close to your infants (e.g. feelings of love, connection, engagement, etc.) including information on what this felt like, why you felt this way, what was happening at the time and who was involved’.* The form also included questions in order to describe the population and timing of the data collection. These included the date when the form was completed, whether they were a mother or a father, the parent’s date of birth (to help identify if parents completed more than one form), the infant’s date of birth and gestational age at time of birth. All parents were also provided with an information sheet about the study. All participant documentation (e.g. information sheet, emotional closeness form) was available in Swedish, English and Finnish.

### Procedure

Staff at the NUs were notified about the study and posters were displayed in the units. In all three countries all parents, irrespective of when their infant had been born were approached by the researchers/NU staff to take part. In Sweden and Finland, the emotional closeness form and information sheet was made available to parents in parent areas in the NUs, e.g. parents’ living room, kitchen and any rooms where parents were situated. In both these settings any parent could complete an emotional closeness form at any point. In England, the ethics committee requested that a signed consent form was obtained from the parent prior to participation. In these occasions, parents were provided with verbal/written information, and then 24 h later they were re-approached to ascertain whether they would like to participate. When signed consent was obtained, copies of the emotional closeness form were provided, to be completed whenever the parent wanted to. In Sweden and Finland, consent for participating in the study and for data to be published was implicit in terms of parents completing the ‘emotional closeness’ form. In all settings, parents were advised to post the completed emotional closeness form into a designated (locked) box on the maternity ward/NU.

### Participants

While all parents of infants cared for in a NU were eligible to participate, parents who did not have sufficient verbal/written Swedish, English or Finnish were not able to take part. Parents of infants whose health was severely compromised were also not actively targeted for recruitment to prevent against additional distress. A total of 23 participants took part in the study: Sweden, n = 8; England, n = 6; Finland, n = 9. In Sweden and Finland, all parents submitted one emotional closeness form. In England one parent submitted 23 forms, one parent submitted three forms and one parent submitted two forms. A total of 48 separate instances/records of emotional closeness were captured during this study.

### Data analysis

All data from the emotional closeness forms were typed into word documents. The Swedish and Finnish data were translated into English together with comments explaining nuances by RF and AA. Data were analyzed separately by each author on an inductive basis using Attride-Stirling’s thematic networks analysis model [21]. The data were read several times to get an overview of the narratives. Meaningful text segments (sentences) were initially organized into different basic themes, and then merged to form organizing themes. In the last step, an overarching super-ordinate (global) theme that encapsulated the principal metaphors in the text was developed. All analytical decisions were discussed and revised between all authors until consensual validation was achieved. In the findings, the letters and numbers refer to the participant’s country: i.e. Swedish (S), English (E) and Finnish (F), who completed the emotional closeness form: i.e. mothers (M) and fathers (F), followed by a numerical code.

## Results

We adopted a salutogenic approach [19, 20] for this study in terms of focussing on parents’ experiences of emotional closeness with their infants. However, it should be recognised that despite the guidance provided to parents, some described emotional struggles and difficulties that had prevented them from feeling emotionally close. For example, some referred to distress due to being unable to touch and be with their infant in the early post-natal period; professionals ‘taking over’ their infant’s care; conflicting/inconsistent care; the emotional turmoil of ‘coping with set backs’ and ‘fear of losing’ or feeling ‘unable to protect’ their infant. These barriers and issues are important considerations. However, due to the fact that they are widely reported in previous literature, and outside the aim of our study, the findings presented focus on how, when and why parents felt emotionally close to their infant.

### Pathways for emotional closeness

**Fig. 1:**
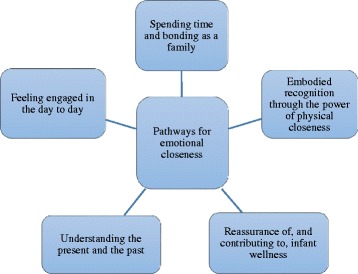
The global theme ‘pathways for emotional closeness’ and the five underpinning organising themes

A global theme of ‘pathways for emotional closeness’ emerged from the data set. This concept relates to how emotional, physical, cognitive and social influences led to embodied feelings of emotional closeness between parents and their infant. The five underpinning organizing themes that explained parents’ feelings of emotional closeness are: Embodied recognition through the power of physical closeness; Reassurance of, and contributing to, infant wellness; Understanding the present and the past; Feeling engaged in the day to day; and Spending time and bonding as a family (Fig. 1).

### Embodied recognition through the power of physical closeness

Mothers and fathers described numerous ways in which emotional closeness was attained through physical closeness; acts that served to provide an embodied recognition of knowing their infants and being known as a parent. For many parents these feelings started when seeing their infant. Parents described how their infants appearance of looking “*helpless”,* “*vulnerable”* and “*small”*, increased their feelings of protectiveness, warmth and love. Parents emphasized the importance of being face-to-face and having eye contact. A mother with an infant born in the 25^th^ week wrote when her son was almost 2 months old: “*Beautiful moments are when the baby is in my lap and looks me into the eyes and I believe him recognizing his mother*” (FM2).

Touching and smelling was reported to have increased parental feelings of emotional closeness as it provided a reality that this was their baby:

“*I went to see the baby after waking* [from the caesarean operation]. *I was able to touch him in the incubator. This was my proof that I had my baby*” (EM1).

Another mother described: “*I then got to kiss her forehead and smell her for the first time. Then I had an evident sensation of she is mine! She smelled MINE!”* (SM5).

Touching was also reported to have had an additional reciprocal value of providing comfort to their infant. Some of the parents described that when they put their hands on their infant’s head or back, the infant relaxed or fell asleep. This is reflected in one of the mother’s descriptions of what happened when her son had a long line inserted into his leg: “*I place a soothing hand on his back and he settles down and goes back to sleep. My boy knows who is mummy is”* (EM1).

Many parents reflected on the impact of holding their infant and/or having their infant ‘kangaroo’ (i.e. skin-to-skin). There were two major influences of holding/kangaroo care on emotional closeness. First, parents were able to provide comfort and security which was displayed in the infant when s/he settled down:

“*Also had kangaroo time with the little one, he slept the whole time and afterward was lifted in the exact same position back in his incubator. I was later told that he was settled all afternoon*” (EM1).

Second, holding/kangarooing was an activity that induced feelings of love and attachment. For many parents, holding the infant and/or being skin-to-skin was the first time they felt their infant was theirs. A father wrote: “*The first time I felt emotional closeness towards the girls was when I got them in kangaroo care*” (FF4). By being physically close the parent-infant bond was strengthened:

*“My feelings towards him got really strong on the second day when I got him into kangaroo care. The closeness has strengthened the bond between us. We are in kangaroo care for 3–6 hours per day”* (FM8)

### Reassurance of, and contributing to, infant wellness

Reassurance of the infant’s wellness was vital for feeling emotionally close for many parents. Parents described how reassurance of their infant’s health and wellbeing was derived from: signs of medical improvements such as weaning from medications; being placed in a cot instead of an incubator; by hearing the first cry straight after delivery; staff putting clothes on the infant for the first time; or removing tubes, cords and machines from the infant:

*“When we moved X to a cot and wheeled him to our room at the neonatal unit, X had no feed tube or any other cables and instruments on him. It just felt SO good!”* (EM5).

Breastfeeding was emphasized by English and Finnish mothers as an activity that made them feel emotionally close to their infants. The impact of breastfeeding was mainly related to the mothers’ unique responsibility and that breastfeeding was “*the only thing*” and “*the best thing*” they could do because of the benefits of breast milk:

*“I started to express as I know this is one of the best things I can do for my baby right now. This was reinforced the next day when he kept down my colostrum but spits/possets all the formula top-up. My body has responded and he has not needed a formula top-up since as I’ve been producing milk in excess of his needs*” (EM1)

Breastfeeding was experienced as a reciprocal act that constituted an evident link between the mother and her infant:

“*The most wonderful moment has probably been when the baby was on the breast for the first time. That feeling that the child gets nourishment from you makes us closer to each other”* (FM7).

### Understanding the present and the past

Parents described the importance of understanding what was happening for feeling emotionally close. Knowing how care was provided (e.g. procedures, technical devices, staff routines), what was expected of them as parents, and understanding the infant’s signals enabled parents to relax and be in the present. The knowledge of their infant’s medical status, gained through the communication with staff and by spending time with their infant, made parents feel more confident in the parental role:

“*During the medical round when the doctor asked, how are your babies doing? I was very proud when I was able to tell them about my observations about the babies”* (FM6).

An awareness and understanding of the parent’s own emotions and experiences was also identified as important for feeling emotionally close. One of the mothers wrote:

*“This is a really difficult question. I don’t know when it happened and when I started to feel it, but it’s likely that it was about 1 week after birth because the first week was difficult with lots of tears and everything that had happened so quickly. So I would say, when everything has calmed down and you’ve had the time to understand what has happened and what you have been through”* (SM3)

### Feeling engaged in the day to day

Parents described pride and joy in providing ‘normal’ care for their infant. Doing simple and ordinary parenting tasks made them feel that that the infant was theirs; changing diapers, putting on clothes and washing and bathing their infant were significant events. One mother stated that she felt emotionally close: “*Especially cares times because I get to physically look after my babies’ needs and bond with my child as a mother*” (EM3). Parents who wrote about caretaking often referred to how these tasks increased their sense of connection and love:

“*When we started to take care of the daughter more and more ourselves, the connection and love became stronger and stronger and still is!*” (SM5).

Some parents also specifically referred to how their increasing involvement in caretaking duties had had a simultaneous influence on their growing sense of commitment and connection:

*“During the following days, the commitment and connection strengthened, especially when I got to spend all three nights at the neonatal unit next to my baby although he was on monitor. I got to take care of him independently and almost according to the baby’s rhythm during the night”* (FM9).

### Spending time and bonding as a family

Many parents reported that being present in the unit and spending time together, parent and infant or the whole family, made them feel emotionally close:

“*It’s always hard to put words on emotions. But when you got to take your babies up and spend time with them for the first time as a family*” (SF7).

Some mothers described feeling proud when watching their partners bond with their infant, as well as when they received encouragement and affirmation from their partners when providing care:

“*I cleaned him and changed him more confidently 2*^*nd*^*time. I did it while his father watched and I felt so, so, proud and for the first time maybe like a mum. My partner was very impressed with me!!!”* (EM2).

Parents in Sweden and Finland highlighted the importance of feeling and being a family when alone with their infant. This was facilitated when parents had their own room on the NU which they could bring the infant into:

“*Yesterday, it was also a wonderful moment when the father came and we were allowed to be alone in the room, as a family, without nurses or other parents”* (FM2).

Bringing the whole family together (i.e. parents, infant and siblings) was often described as momentous:

*“To see the big brother is big. Maybe most for the mum. Such a mix of emotions bubbles up. The longing for him that has grown more intense for each day that we have been apart. The pride I feel when he comes towards me. Total happiness when he curiously gets into bed with me and looks at his little sister. I feel more whole than ever. My family, together”* (SM2).

## Discussion

Our findings highlight that parents experienced different, and at times multiple, pathways for feeling emotionally close to their infant. These pathways of emotional, physical, cognitive and social influences led to embodied feelings of parent-infant emotional closeness. We identified five important areas comprising significant events/actions/processes that facilitated and contributed to the feeling of emotional closeness: Embodied recognition through the power of physical closeness; Reassurance of, and contributing to, infant wellness; Understanding the present and the past; Feeling engaged in the day to day; and Spending time and bonding as a family.

These findings are, in one way unique, as to date no other study has specifically explored experiences of emotional closeness in parents of infants in NUs. Unlike parents of healthy and term infants, parents of preterm and newborn ill infants spend their early days together in a highly medical context, where the ‘ownership’ of the infant could be debateable [3, 22, 23]. Because of the infant’s medical demands, professional skilled care is needed and the ‘normal’ parental role may be reduced [3, 24, 25]. Furthermore, a disruption of the family, where parent-infant separation is commonplace, is a clear demarcation and signal that parents are not needed by their infant [26]. Subsequently, parents of infants who need neonatal care, unlike those of term infants, start their journey from a subordinate position. Moreover, while all new parents can experience anxiety, parents of premature infants often have higher levels of stress and depression [27] due to real concerns over the infant’s viability. In our study, through adopting a salutogenic approach, we were able to identify that parents were able to develop different pathways to develop emotional closeness with their infants, despite the stress and challenges faced. These insights may help health professionals to identify and support the developing parent-infant relationship.

Numerous studies have shown the importance of physical closeness on parental well-being [2, 28], and in the last decade research has focused on and demonstrated positive effects of skin-to-skin contact (i.e. kangaroo care) on parent-infant outcomes [29, 30]. Feeley and colleagues [28] described the importance of contact such as touch for fathers to be able feel the joy of parenting. This supports our findings in which parents described seeing the infant, having eye-contact, touching and holding as significant experiences for feeling emotionally close.

Reassurance of the infant’s health and wellbeing also appeared to be vital for these parents. These insights thereby suggest that having faith and trust for the viability of the infant promotes parents’ feelings of emotional closeness. This does not mean that parents whose infant is terminally ill would not feel emotionally close, but that it is much harder and may take a longer time [3, 31]. Previous studies have found that when parents believe that their infant might die, they distance themselves from the infant, geographically and emotionally [3, 25]. Ongoing reassurance by staff on the infant’s clinical progress therefore appears crucial.

Another pathway to closeness involved parents performing day to day activities of their infant’s care. Our findings showed that feeling engaged was the result of providing ‘normal’ care for their infant and experiencing it as joyful. Furthermore, by being engaged, parents also experienced a growing sense of commitment. Indeed, Winnicott [32] stated *“The mother’s pleasure has to be there or else the whole procedure is dead, useless, and mechanical”* (p. 27). On a busy NU, staff members may forget that basic caretaking roles such as putting clothes on an infant for the first time is a significant and primary parental role. Our findings are in concordance with other studies, which show that parents need to be present on the neonatal unit, involved and feel that they are in charge of such activities [18, 33].

Our findings also showed the importance of how parent’s need to have an understanding and an awareness of their own experience and emotions in order to feel emotionally close. Parents often face surreal, chaotic and deeply distressing situations in the NU and the combination of parental issues and infant vulnerabilities can be especially difficult. Parents are not mentally prepared for the experience and may put their “life on hold”. This represents a strategy where they block their emotions and endure the situation with consequences of alienation – in regard to their own needs, from their infants or from being parents [9, 25]. A study by Coppola et al. [34] reported that more than 80 % of the parents felt the need to share their experiences during the time at a NU. However, parents might avoid disclosing and sharing experiences with others because of feelings of social isolation, guilt, shame and thinking that others do not understand. Hence, more research is needed in order to identify feasible and effective interventions that support parents emotionally, e.g. parent support groups [4, 35, 36]. Furthermore, the interpersonal relationships with staff are of crucial concern for feeling valued, respected and involved [33]. One feasible approach to enable staff to support parents emotionally is to facilitate person-centred communication (PCC). McCormack and colleagues [37] have defined PCC as a model of care, which includes components such as: building mutual trust and understanding; treating the person as an individual; respecting the rights of the person, sharing decision-making, providing holistic care and developing therapeutic relationships. Within neonatal care, a randomised controlled trial was conducted to explore the effects of PCC on parental stress. The quantitative analyses showed no difference in terms of parental stress [38]. However, the qualitative evaluation of the study showed that the components of the intervention (i.e. scheduled nurse-parent dialogues, semi-structured reflection sheets for parents to fill out and PCC) enabled the parents to discover and express emotions, gain a deeper form of communication and experience a mutual understanding (i.e. nurses gaining insights into the lives of the parents and parents understanding each other) [39].

An interesting finding in our study was that only the English and Finnish mothers described how breastfeeding facilitated feelings of emotional closeness. As this is a qualitative study and women were provided with an open brief to describe moments/experiences of closeness it may be that Swedish mothers did not consider breastfeeding to be such a significant act, or that they did not write about it. The lack of consideration of breastfeeding may also be contextual. The Swedish unit had a single room design and the English and Finnish units had open bay rooms. In a single room unit, staff may be less inclined to promote breastfeeding, as parents spend all their time with their infants. A previous study [40] showed that when parents had the opportunity to stay day and night with their infant, the ‘necessity’ of breastfeeding for becoming ‘a good enough mother’ attenuated.

Spending time together as a family was described by mothers and fathers as important for emotional closeness. Family Centred Care (FCC) is a philosophy, comprising a set of values, policies and approaches to services for infants and children with special needs and their families. The FCC approach places parents at the centre of care-giving processes and procedures [41, 42]. However, ‘parents’ often become synonymous with ‘mothers’, especially in healthcare of newborn infants. Internationally, there are wide variations in mothers and fathers presence in the NUs; fathers visit less and for a shorter time than mothers, and are less engaged in caretaking (e.g. infant cleaning and feeding) [26, 43]. Efforts need to be made to ensure that the design of the unit, as well as staff support of parental presence and engagement, enable both fathers and mothers to participate on equal terms. Furthermore, a ‘family’ may include many significant people (e.g. partners, siblings, grandparents) who are vital for parents’ emotional wellbeing. A design and philosophy in NUs where parents and the family are truly at the centre of care may be an important step to increase emotional closeness.

A potential limitation of the study is that there may have been an ‘elite-bias’; mothers and fathers who were more confident in expressing emotions in writing participated. However, the writing style and the length of ‘stories’ differed substantially. Furthermore, it could be that the risk for an ‘elite-bias’ is reduced when parent write their stories rather than tell them, especially when anonymous. Some parents completed the emotional closeness form on the first day after birth, while others completed the form two months after birth. This could reflect when the parents were recruited/invited to participate in the study and/or simply when parents wanted to. Regardless, from the insights captured, we believe that parents highlighted experiences that were prominent for them, a positive critical point during the hospital stay, regardless of when the event(s) occurred. To enhance credibility and trustworthiness [44]. Swedish and Finnish stories were translated into English with comments explaining nuances and all authors were involved in the coding and analysis. While socio-demographic, cultural, NU design and policy differences may limit the transferability of the findings, similar issues were raised by parents from different countries and settings and who had infants of different gestational ages at birth. Further, more in-depth qualitative research (i.e. using interviews and/or focus groups) across different international contexts and with different population groups would help to authenticate the findings generated. Very rarely do researchers studying parenting of preterm infants write about joy and happiness. Hypothetically, more research with a salutogenic approach and/or on positive emotions would be beneficial and shed light on how to make improvements within neonatal care.

## Conclusion

In this study we found that despite the stress and adversities faced, different pathways comprising cognitive, physical emotional and social influences, facilitated parental feelings of emotional closeness to their infants in the neonatal unit. These cross-national insights highlight the significance of close physical contact through parents seeing, touching, holding and being in skin-to-skin contact with their infants. Parents need ongoing reassurance and clear and comprehensible information about their infant’s progress, to have opportunities to understand their own experiences and emotions, to be engaged in basic care-giving activities and for units to accommodate and enable time alone as a family. While further salutogenic-focused research to substantiate the findings is needed, the insights emphasise how health care staff can create a climate where parents’ emotions and their emotional journey are supported.

## Abbreviations

FCC, family centred care; NU, Neonatal Unit; PCC, person centered communication
